# Specificity of Polygenic Scores for Psychiatric Disorders Beyond Transdiagnostic Genetic Risk

**DOI:** 10.1001/jamanetworkopen.2025.48518

**Published:** 2026-01-08

**Authors:** Engin Keser, Wangjingyi Liao, Andrea G. Allegrini, Thalia C. Eley, Kaili Rimfeld, Margherita Malanchini, Robert Plomin

**Affiliations:** 1 Social, Genetic and Developmental Psychiatry Centre, King’s College London, London, United Kingdom; 2 School of Biological and Behavioural Sciences, Queen Mary University of London, London, United Kingdom; 3 Department of Psychology and Language Science, University College London, London, United Kingdom; 4 Department of Psychology, Royal Holloway University of London, London, United Kingdom

## Abstract

**Importance:**

Polygenic scores (PGSs) are increasingly used to estimate genetic liability for psychiatric disorders, but their limited disorder specificity constrains their clinical and research utility.

**Objective:**

To test whether associations between psychiatric PGSs and psychiatric outcomes are primarily driven by transdiagnostic or disorder-specific genetic liability.

**Design, Setting, and Participants:**

This cohort study used a population-based sample from the Twins Early Development Study of twins born in England and Wales between January 1994 and December 1996. Mental health data were collected from July 2021 through June 2023, when participants were aged 25 to 28 years. The analyses include participants who had available genetic data and at least 1 quantitative symptom score.

**Main Outcomes and Measures:**

The main outcomes were quantitative symptom scores and self-reported psychiatric diagnoses. Associations were tested using generalized estimating equations with 3 types of PGSs: (1) uncorrected; (2) a transdiagnostic score indexing the shared genetic liability across 11 major psychiatric disorders (general factor of psychopathology [p]); and (3) residual disorder-specific scores corrected for p (non-p).

**Results:**

Analyses included 6567 participants (mean [SD] age, 26.4 [0.93] years; 4220 female [64.3%]). For quantitative symptom scores, the PGS for p showed comparable or larger associations with outcomes than the corresponding uncorrected PGSs across most symptom domains. For example, the effect size for the association between the p PGS and posttraumatic stress disorder (PTSD) symptoms was 0.15 (95% CI, 0.11-0.18) compared with 0.10 (95% CI, 0.07-0.13) for the uncorrected PTSD PGS; associations for other traits were smaller in magnitude but directionally consistent. Associations with self-reported diagnoses were of similar magnitude for the p and uncorrected PGSs. Some disorder-specific (non-p) PGSs retained significant trait-specific associations, including with symptom scores, such as problematic alcohol use (β = 0.09; 95% CI, 0.07-0.12) and PTSD (β = 0.08; 95% CI, 0.05-0.11), and with self-reported diagnoses, such as PTSD (odds ratio, 1.43; 95% CI, 1.18-1.74) and anorexia nervosa (odds ratio, 1.28; 95% CI, 1.03-1.59), suggesting residual disorder specificity. Most uncorrected PGSs showed extensive cross-trait associations, which were substantially attenuated after accounting for p, suggesting that much of the genetic signal was associated with transdiagnostic genetic liability.

**Conclusions and Relevance:**

This cohort study found that the observed associations between PGSs and psychopathology outcomes were largely explained by transdiagnostic genetic liability, with some disorder-specific signals also detected. Accounting for shared genetic effects may enhance specificity of PGSs, while transdiagnostic scores may inform risk stratification and interventions targeting general psychopathology.

## Key Points

**Question:**

To what extent do polygenic scores for psychiatric disorders index transdiagnostic rather than disorder-specific genetic risk?

**Findings:**

In this cohort study of 6567 young adults in the UK, the polygenic score showed at least comparable associations with psychiatric outcomes compared with the corresponding uncorrected polygenic scores. Disorder-specific polygenic scores showed attenuated associations, although a small number retained significant disorder-specific risks.

**Meaning:**

These findings suggest that polygenic scores may primarily reflect genetic liability shared across psychiatric disorders, emphasizing the need to disentangle transdiagnostic and disorder-specific signals to improve interpretability and clinical relevance.

## Teaser

This cohort study examines the extent to which polygenic scores for psychiatric disorders index transdiagnostic general factor for psychopathology vs disorder-specific risk among young adults in the UK.

## Introduction

Genome-wide association studies (GWASs) have transformed our understanding of the genetic architecture of psychiatric disorders.^1^ In addition to identifying replicable genetic loci associated with disorders, summary statistics from GWASs have made it possible to construct polygenic scores (PGSs), which aggregate the effects of thousands of single nucleotide variants identified in a GWAS to estimate an individual’s genetic liability for a given trait or disorder.^2^

Despite the increasing use of PGSs in psychiatric research,^3^ their clinical utility remains limited.^4^ One major constraint is their modest explanatory power, as PGSs typically explain only a small proportion of phenotypic variance in target outcomes. This limitation stems partly from underpowered GWAS sample sizes, unmeasured genetic variants (eg, rare variants), phenotypic heterogeneity, and imprecise trait definitions. Another major limitation is the lack of specificity in PGSs for the disorders for which they were derived. Polygenic scores show transdiagnostic associations with multiple psychiatric outcomes, reflecting substantial genetic overlap across disorders.^5,6,7^ Although pairwise genetic correlations across disorders are substantial, they remain well below 1.0, suggesting both shared and unique genetic contributions. However, the extent to which PGSs capture disorder-specific vs transdiagnostic genetic liability remains unclear.

One approach to investigating genetic specificity is to create PGSs corrected for transdiagnostic effects. Shared variance across psychiatric disorders is often conceptualized as a general factor of psychopathology (p), which represents broad liability to multiple mental health conditions.^8,9,10^ Although initially identified at the phenotypic level, the p-factor has since been observed in genetic and genomic analyses.^11,12,13,14^

In prior work,^13^ we used genomic structural equation modeling (genomic SEM)^15^ to decompose the genetic variance across 11 major psychiatric disorders into a transdiagnostic factor (p) and disorder-specific residual components. This genomic SEM analysis yielded summary statistics for both the shared and unique genetic components (referred to as non-p to represent residual genetic variance independent of shared liability).

In this study, we used these summary statistics to construct PGSs in a UK population–based cohort of young adults from the Twins Early Development Study (TEDS). We tested associations between quantitative symptom scores and self-reported diagnoses and 3 types of PGSs: uncorrected PGSs, a transdiagnostic PGS indexing shared genetic liability across 11 disorders (p), and disorder-specific PGSs corrected for shared variance (non-p). By comparing these PGSs, we aimed to investigate the extent to which associations with psychiatric outcomes reflect transdiagnostic versus disorder-specific genetic liability.

## Methods

### Sample

This cohort study was preregistered with the Open Science Framework prior to accessing the data.^16^ Participants were drawn from TEDS,^17^ a longitudinal cohort of 13 759 twin pairs (27 518 individuals) born in England and Wales between January 1994 to December 1996. The TEDS has ethical approval from the King’s College London Research Ethics Committee, and written informed consent was obtained from all participants. Our study reports a secondary analysis of preexisting data with all identifying information removed by the TEDS team. Under King’s College London research ethics guidance on the analysis of preexisting data, secondary analyses do not require additional ethical approval. This study followed the Strengthening the Reporting of Observation Studies in Epidemiology (STROBE) reporting guideline.

The original cohort was representative of the UK population in terms of ethnicity and socioeconomic status,^18^ which was largely maintained at age 26 years, despite some attrition.^17^ Ethnicity was reported by parents at first contact and by participants at age 26 years using UK census–based categories. Ethnicity was not analyzed due to the predominantly White sample (94%), which limited statistical power for minority groups.^17^ Mental health data were collected between July 2021 through June 2023, when participants were aged 25 to 28 years, via self-report questionnaires administered online or by mail.

Genotyping was conducted on 2 different platforms (Affymetrix GeneChip 6.0 [Thermo Fisher Scientific] and HumanOmniExpressExome-8 v1.2 [Illumina]). Details on DNA collection, genotyping, and quality control are described elsewhere.^14,19^ Individuals with both genetic data and at least 1 quantitative symptom score were included in the study. Final analytic samples included a range of individuals (including dizygotic twins), depending on the outcome measure.

### Measures

#### Quantitative Symptom Measures

Symptom data were available for 8 psychiatric disorders (Table).^22,23,24,25,26,27^ Descriptions of each measure are provided in the eMethods in Supplement 1, with the descriptive data presented in eTable 1 in Supplement 2.

**Table.** Quantitative Symptom Measures Used in This Study

Symptoms (related disorder)	Measure	Source
Anxiety symptoms (anxiety disorder)	Generalized Anxiety Disorder– Dimensional	Lebeau et al,^20^ 2012
Attention-deficit symptoms (ADHD)	Conners Rating Scale	Conners,^21^ 2008
Autistic traits (ASD)	Ritvo Autism and Asberger Diagnostic Scale	Eriksson et al,^22^ 2013
Depressive symptoms (MDD)	Moods and Feelings Questionnaire	Angold et al,^23^ 1995
Mania/hypomania symptoms (bipolar disorder)	Mood Disorder Questionnaire	Hirschfeld et al,^24^ 2000
Alcohol use (problematic alcohol use disorder)	Alcohol Use Disorder Identification Test	Saunders et al,^25^ 1993
PTSD symptoms (PTSD)	Posttraumatic Stress Disorder Checklist 6	Weathers et al,^26^ 1993
Psychotic experiences (schizophrenia)^a^	Specific Psychotic Experiences Questionnaire: Hallucinations and Paranoia subscales	Ronald et al,^27^ 2014

Abbreviations: ADHD, attention-deficit/hyperactivity disorder; ASD, autism spectrum disorder; MDD, major depressive disorder; PTSD, posttraumatic stress disorder.

^a^ Symptoms related to schizophrenia were assessed using dimensional measures of psychotic-like experiences, including hallucinations and paranoia.

#### Self-Reported Diagnoses

Self-reported diagnostic data were available for 9 disorders: attention-deficit/hyperactivity disorder (ADHD), anorexia nervosa, anxiety disorder, autism spectrum disorder (ASD), bipolar disorder, major depressive disorder (MDD), obsessive-compulsive disorder, psychosis or psychotic illness (excluding schizophrenia), and posttraumatic stress disorder (PTSD). Participants were provided with a list of mental health conditions and asked whether they had ever received a professional diagnosis, regardless of their status at the time of data collection. Responses were recorded as yes or no. Frequencies are presented in eTable 2 in Supplement 2. Because of a lack of participants with schizophrenia, psychosis or psychotic illness was used as a proxy in analyses involving the schizophrenia PGS.

#### GWAS Summary Statistics

We used publicly available summary statistics from GWASs of 11 major psychiatric disorders: ADHD,^28^ schizophrenia,^29^ anxiety disorder,^30^ ASD,^31^ bipolar disorder,^32^ MDD,^33^ PTSD,^34^ Tourette syndrome,^35^ problematic alcohol use,^36^ obsessive-compulsive disorder,^37^ and anorexia nervosa.^38^ All GWASs were based on case-control designs, except for problematic alcohol use, which used a symptom-level measure of alcohol use. Details are provided in eTable 3 in Supplement 2. Using genomic SEM, we derived summary statistics for a transdiagnostic factor (hereafter, p) and residual disorder-specific components (hereafter, non-p).^13^ These summary statistics were used in this study to construct PGSs.

#### PGS Construction in TEDS

Polygenic scores were constructed in the TEDS sample using LDpred2^39^ with the auto option and default parameters. Analyses were restricted to HapMap3 variants^40^ and based on precomputed linkage disequilibrium matrices from the UK Biobank.^40^ Both the discovery GWASs and the TEDS sample were limited to individuals of European ancestry.

### Statistical Analysis

We used generalized estimating equation regressions with an exchangeable correlation structure to account for relatedness within the sample. Gaussian models were used for continuous outcomes and binomial models for binary outcomes, implemented using R, version 4.4.2 (R Foundation for Statistical Computing) package gee.^41^ All statistical tests were 2-sided with a significance threshold of *P* < .05. False discovery rate correction was applied to account for multiple testing.^42^ Continuous outcomes were residualized for age and sex and standardized prior to analysis. All models included the top 10 ancestry principal components, genotyping batch, and genotyping chip as covariates.

We first tested associations between each PGS and its corresponding quantitative symptom score and self-reported diagnosis. Associations with continuous outcomes were summarized using standardized regression coefficients (β), and associations with binary outcomes were summarized using odds ratios (ORs) with 95% CIs. To assess pleiotropy, we examined cross-trait associations between uncorrected and non-p PGSs and symptom scores for noncorresponding phenotypes (eg, associations between the MDD uncorrected and non-p PGSs and PTSD symptom score). Analyses were repeated after excluding individuals with self-reported diagnoses to examine whether associations were primarily driven by these individuals.

Additional, non-preregistered sensitivity analyses were conducted to test the robustness of our findings. First, to characterize the phenotypic profiles of participants with an elevated p PGS, we compared symptom severity and diagnostic burden in individuals in the top 1% of the p PGS distribution with those in the top 1% of the uncorrected and non-p PGS distributions. Diagnostic comorbidity was defined as the number of self-reported psychiatric diagnoses endorsed by each participant. Second, to assess the performance of PGSs at clinically meaningful levels of symptom severity, we examined associations with high-risk status (defined using clinical cutoffs where available) and compared the ability of each PGS to distinguish individuals in the top vs bottom deciles of symptom distributions (eMethods in Supplement 1). Relative performance of PGSs was evaluated by comparing effect sizes across models.

## Results

### Associations Between PGSs and Corresponding Symptom Scores

**Figure 1.**
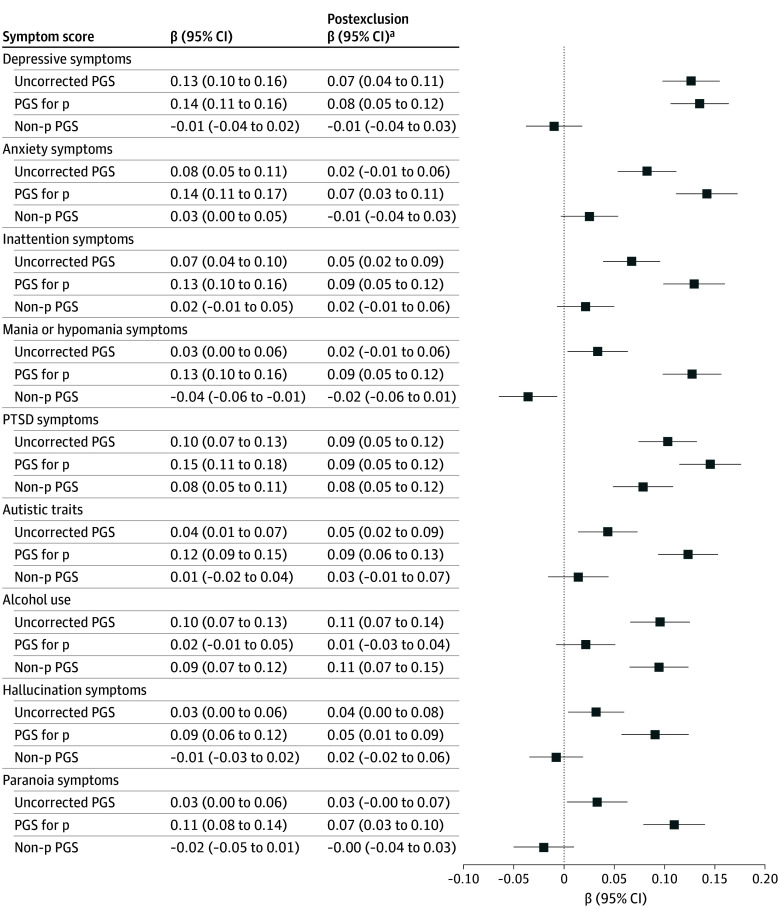
Associations Between Polygenic Scores (PGSs) and Corresponding Quantitative Symptom Scores All variables were residualized for age and sex and standardized prior to analysis. Models were additionally adjusted for the first 10 genetic principal components, as well as genotyping chip and batch. Exact estimates and *P* values are reported in eTable 4 in Supplement 2. p Indicates transdiagnostic general factor of psychopathology; PTSD, posttraumatic stress disorder. ^a^Postexclusion estimates (excluding participants with any self-reported diagnoses) are displayed but not plotted. Exact estimates and *P* values are provided in eTable 5 in Supplement 2.

The analytic sample included 6567 participants (mean [SD] age, 26.4 [0.93] years, 4220 female [64.3%] and 2347 male [35.7%]). Figure 1 presents the associations between each PGS and its corresponding quantitative symptom score. Four key findings emerged. First, all uncorrected PGSs were significantly associated with their corresponding symptom scores, although effect sizes were modest. Second, non-p PGSs showed attenuated associations compared with uncorrected PGSs and were predominantly nonsignificant. Exceptions were observed for alcohol use (β = 0.09; 95% CI, 0.07-0.12; *P* = 6.14 × 10^−10^) and PTSD (β = 0.08; 95% CI, 0.05-0.11; *P* = 4.67 × 10^−7^), indicating a residual disorder-specific genetic signal. Third, the p PGS was significantly associated with all symptom scores except alcohol use, ranging from a β coefficient of 0.09 (95% CI, 0.06-0.12; *P* = 2.28× 10^−7^) for hallucination symptoms to 0.15 (95% CI, 0.11-0.18; *P* = 4.14 × 10^−19^) for PTSD symptoms. In 5 of 9 comparisons, the p PGS outperformed the uncorrected PGSs and performed comparably in the remaining 4. Fourth, these patterns remained consistent after excluding individuals with self-reported diagnoses, although some associations were attenuated and no longer statistically significant. The full results are presented in eTables 4 and 5 in Supplement 2.

### Associations Between PGSs and Corresponding Self-Reported Diagnoses

**Figure 2.**
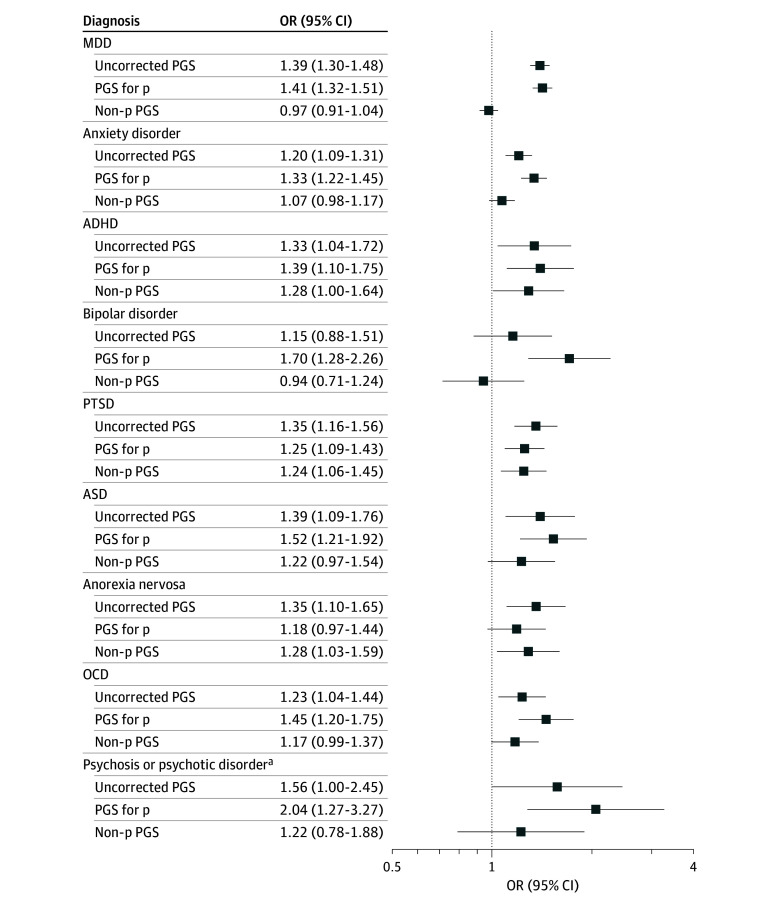
Associations Between Polygenic Scores (PGSs) and Self-Reported Diagnoses All models were adjusted for the first 10 genetic principal components, as well as genotyping chip and batch. Exact estimates and *P* values are provided in eTable 6 in Supplement 2. ADHD indicates attention-deficit/hyperactivity disorder; ASD, autism spectrum disorder; MDD, major depressive disorder; OCD, obsessive-compulsive disorder; OR, odds ratio; PTSD, posttraumatic stress disorder. ^a^Psychosis or psychotic illnesses in relation to the PGS for schizophrenia.

Figure 2 presents the ORs for the associations between each PGS and its corresponding self-reported diagnosis (details provided in eTable 6 in Supplement 2). Despite the relatively small number of participants with self-reported diagnoses, 7 of 9 uncorrected PGSs were significantly associated with their respective diagnoses. Notably, the non-p PGSs for anorexia nervosa (OR, 1.28; 95% CI, 1.03-1.59) and PTSD (OR, 1.24; 95% CI, 1.06-1.45) also retained significant associations, indicating residual disorder-specific genetic variance. The p PGS showed associations of comparable magnitude to the uncorrected PGSs but did not outperform them.

### Cross-Trait Associations Between PGSs and Symptom Scores

**Figure 3.**
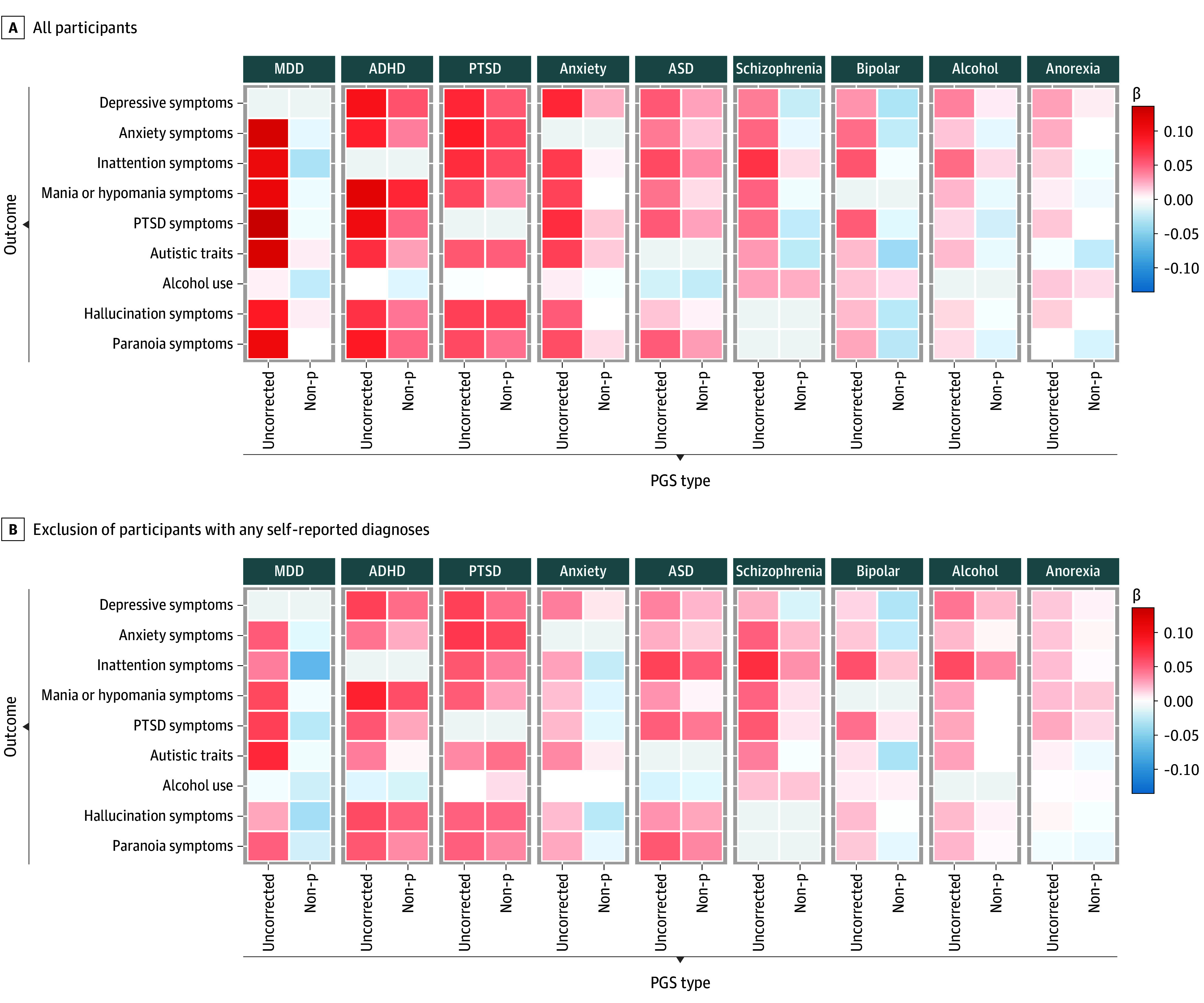
Cross-Trait Associations Between Polygenic Scores (PGSs) and Quantitative Symptom Scores Heat maps display standardized regression coefficient (β) estimates for associations between each PGS and noncorresponding symptom domains (eg, major depressive disorder [MDD] PGS with anxiety symptoms). Within each facet, columns compare uncorrected and non-p PGSs. Colors encode standardized β estimates on a symmetric scale centered around 0 (intensity reflects magnitude). All variables were residualized for age and sex and standardized prior to analysis. Models were additionally adjusted for the first 10 genetic principal components, as well as genotyping chip and batch. Exact estimates and *P* values are reported in eTable 7 (for the full sample) and eTable 8 (for the postexclusion sample) in Supplement 2. ADHD indicates attention-deficit/hyperactivity disorder; ASD, autism spectrum disorder; PTSD, posttraumatic stress disorder.

As shown in Figure 3, the cross-trait analyses (details in eTable 7 in Supplement 2) revealed extensive pleiotropy. Several uncorrected PGSs, including those for anxiety disorder, MDD, schizophrenia, PTSD, and ADHD, showed widespread associations with multiple noncorresponding symptom scores. In many cases, these cross-trait associations were of similar or greater magnitude compared with same-trait associations. For example, the MDD PGS was similarly associated with symptoms of MDD (β = 0.13; 95% CI, 0.10-0.16; *P* = 2.09 × 10^−17^), ASD (β = 0.13; 95% CI, 0.10-0.16; *P* = 1.53 × 10^−14^), and PTSD (β = 0.14; 95% CI, 0.11-0.17; *P* = 1.41 × 10^−16^). An exception was the problematic alcohol use PGS, which was associated only with symptoms of ADHD (β = 0.05; 95% CI, 0.02-0.08; *P* = .007) and MDD (β = 0.04; 95% CI, 0.01-0.07; *P* = .02). Accounting for transdiagnostic variance substantially attenuated most associations, indicating that much of the observed cross-trait signal was driven by transdiagnostic genetic liability. Similar, though attenuated, patterns were observed after excluding participants with self-reported diagnoses (eTable 8 in Supplement 2).

### Sensitivity Analyses

Sensitivity analyses examined the phenotypic profiles of individuals at the high end of the p PGS distribution and evaluated PGS performance at clinically relevant symptom levels and across the extremes of the symptom distributions. First, participants in the top 1% of the p PGS distribution showed elevated symptom levels across multiple domains and higher diagnostic comorbidity compared with those in the top 1% of the uncorrected and non-p PGS distributions (eFigure 1 in Supplement 1), consistent with broad phenotypic expression of transdiagnostic liability. Second, the p PGS and uncorrected PGSs performed similarly in identifying participants with clinically meaningful symptom levels, showing larger associations with high-risk status (eFigure 2 in Supplement 1). Third, the p PGS and uncorrected PGSs showed comparable performance in distinguishing individuals in the top vs bottom deciles of symptom severity (eFigure 3 in Supplement 1). Non-p PGSs generally showed smaller associations, with the exception of problematic alcohol use and PTSD. Full results are provided in the eResults in Supplement 1 and eTables 9 to 11 in Supplement 2.

## Discussion

In this population-based cohort study of young adults, we investigated whether PGSs for major psychiatric disorders index transdiagnostic vs disorder-specific genetic liability. Using both dimensional (symptom scores) and categorical (self-reported diagnosis) outcomes, we compared 3 types of PGSs: uncorrected PGSs, a transdiagnostic PGS (p), and disorder-specific PGSs corrected for transdiagnostic effects (non-p). Overall, associations between PGSs and psychiatric outcomes were largely driven by transdiagnostic genetic risk, with limited evidence for disorder-specific associations. To our knowledge, this study is the first to systematically compare the different types of PGSs across multiple psychiatric phenotypes using both dimensional and diagnostic outcomes.

Our findings highlight the importance of accounting for shared genetic liability when interpreting associations between PGSs and psychiatric phenotypes. The p PGS was consistently associated with symptom scores, often outperforming uncorrected PGSs for corresponding symptom scores. Cross-trait analyses revealed widespread pleiotropy, and adjusting for the p substantially attenuated associations between PGSs and noncorresponding symptom scores. These results suggest that many associations observed with uncorrected PGSs may reflect a general transdiagnostic liability rather than disorder-specific effects, consistent with the substantial overlap in the genetic architecture of psychiatric disorders.^5,7,12,13^ Without accounting for this shared variance, associations between PGSs and psychiatric outcomes may be misinterpreted as disorder specific.

Although the p PGS benefits from increased statistical power through the aggregation of GWAS data across 11 disorders, power alone does not explain the observed associations. Rather, the extent to which a PGS indexes transdiagnostic vs disorder-specific risk may depend, in part, on the phenotypic granularity of the discovery GWAS.^43^ Genome-wide association studies based on narrowly defined or dimensional phenotypes tend to yield greater specificity,^43,44^ which our study found for problematic alcohol use. The problematic alcohol use PGS, derived from an item-level GWAS^36^ and assessed using the same measure in our sample, was the only PGS (both uncorrected and non-p) that was almost exclusively associated with its corresponding phenotype. Notably, it was also the only domain for which the p PGS did not show a significant association. This finding may reflect both the phenotypic specificity captured by the discovery GWAS and the observable nature of problematic alcohol use–related behaviors (eg, failing to complete a task due to drinking).

While the p PGS showed large associations than uncorrected PGSs with quantitative symptom scores, its associations with self-reported diagnoses were broadly comparable to those of uncorrected PGSs. If performance was driven solely by statistical power, similar advantages would be expected across both types of outcomes. Instead, the comparable performance of the p and uncorrected PGSs for diagnostic outcomes suggests that uncorrected PGSs retain some disorder-specific signal, which may be due to their derivation from case-control GWASs that align more closely with diagnostic classifications. In contrast, the larger associations between the p PGS and symptom scores may reflect the p PGS’s closer alignment with dimensional, transdiagnostic measures that capture overlapping symptomatology across disorders. Consistent with this interpretation, when symptom scores were dichotomized to approximate clinical thresholds, we found that the performance of the p and uncorrected PGSs converged, suggesting that uncorrected PGSs may better capture disorder-specific risk in more clinically relevant contexts.

Despite the dominance of transdiagnostic associations, some non-p PGSs retained significant trait-specific associations. For example, the non-p PGS for PTSD was modestly, but robustly associated with both PTSD symptoms and diagnoses, despite only 38% of its genetic variance being independent of the p.^13^ This finding suggests that while shared liability may account for much of the observed genetic signal across psychiatric outcomes, unique disorder-specific risk may remain detectable, particularly in cases such as PTSD, in which the phenotype may represent a biologically cohesive construct.^45^ As GWAS sample sizes increase and phenotyping becomes more refined, these residual disorder-specific signals may become more informative. Emerging methods have offered promise for enhancing specificity of PGSs.^46,47^ In the meantime, PGSs derived from p-corrected summary statistics, as used in this study, offer a practical approach for investigating specificity.

Our findings of modest and nonspecific associations of uncorrected PGSs with psychopathology symptoms are aligned with prior research in population samples^48,49,50^ and further suggest that genetic risk factors underlying clinical diagnoses may also contribute to corresponding symptom dimensions in the general population. This finding aligns with dimensional frameworks, such as Research Domain Criteria^51^ and Hierarchical Taxonomy of Psychopathology,^52^ which conceptualize psychopathology along a continuum of severity rather than as discrete categories. These frameworks better reflect the shared symptomatology and polygenic architecture of psychiatric traits. Quantitative symptom measures, which capture variation across the full spectrum of severity, may therefore be particularly well suited to capturing transdiagnostic risk. When combined with a transdiagnostic PGS, such as the p PGS, they may support population-level risk stratification^52,53,54^ and inform the development of transdiagnostic interventions.^55^

### Limitations

The findings should be interpreted in the context of several limitations. First, reliance on self-report data may introduce recall or reporting biases.^56^ The use of composite symptom scores may also oversimplify the complexity of psychopathology. Alternative approaches, such as network analysis, could better capture the complexity of mental health problems.^57,58^ Second, analyses were limited to individuals of European ancestry and a younger sample, limiting the generalizability of findings to other ancestral populations and age groups. Third, although modest in size, the TEDS sample provides rich phenotypic data closely aligned with the GWAS traits used in our prior work.^13^ Quantitative symptom scores were available for 8 of the 11 disorders and self-reported diagnoses for 9, enabling comparisons of different types of PGSs across both dimensional and categorical outcomes. Fourth, there was a lack of replication in an independent sample. Because PGS associations often have modest effect sizes, particularly in community samples, the absence of replication warrants caution in generalizing these findings. Future research would benefit from replicating and extending these results in larger and more diverse populations with similarly detailed measures.

Finally, the p and non-p PGSs were based on genomic SEM–derived summary statistics.^13^ While SEM is a powerful framework for modeling complex data, the resulting factors are not causal entities^59^ and are sensitive to the traits included in the model.^60^ Consequently, p and, by extension, non-p reflect the structure and limitations of the underlying GWASs, including phenotype definitions and ascertainment strategies.^44,61^ For example, the inclusion of individuals with comorbid diagnoses in discovery GWASs may inflate genetic correlations,^62^ potentially amplifying the p. However, prior simulations showed that extremely high rates of diagnostic overlap and misclassification would be required to account for the observed genetic overlaps.^5^ Additionally, non-p PGSs were constructed by statistically removing p-related variance, but this process is necessarily imperfect. Therefore, the p-factor should not be interpreted as a pure index of general psychopathology but rather as a data-driven summary of genetic covariance across GWASs. Similarly, non-p PGSs approximate residual trait-specific liability rather than represent pure indicators of specificity. Therefore, the p and non-p PGSs used in our study offer a complementary approach to current GWAS methods for investigating specificity and transdiagnostic genetic liability.

## Conclusions

This cohort study using both dimensional (symptom scores) and categorical (self-reported diagnosis) outcomes found that PGSs derived from current GWASs of major psychiatric disorders primarily captured transdiagnostic genetic liability, with limited evidence of disorder specificity. Accounting for this shared liability may enhance the specificity and interpretability of PGSs for specific disorders, while transdiagnostic PGSs, such as the p PGS, may serve as useful tools for population-level risk stratification and inform transdiagnostic interventions.

## Notes

Supplement 1.
eMethods.
**eFigure 1.** Isolating Transdiagnostic Genetic Effects Across 11 Psychiatric Disorders
eResults.
**eFigure 2.** Heat Map of Standardized Symptom Scores in the Top 1% of Each Polygenic Score Distribution **eFigure 3.** Odds Ratios (ORs) for High-Risk Group Status by Polygenic Scores **eFigure 4.** Odds Ratios (ORs) for Top vs Bottom Symptom Deciles by Polygenic Score
eReferences.

Supplement 2. **eTable 1.** Descriptive Statistics of Disorder-Related Symptom Scores Among Genotyped Individuals **eTable 2.** Self-Reported Diagnoses Among Genotyped Participants **eTable 3.** Contributing GWAS Cohorts for the 11 Major Psychiatric Disorders **eTable 4.** Associations Between Polygenic Scores and Corresponding Quantitative Symptom Scores **eTable 5.** Associations Between Polygenic Scores and Corresponding Quantitative Symptom Scores, Excluding Participants With Any Self-Reported Diagnosis **eTable 6.** Associations Between Polygenic Scores and Corresponding Self-Reported Diagnoses **eTable 7.** Cross-Trait Associations Between Polygenic Scores and Quantitative Symptom Scores Beyond Their Primary Phenotype **eTable 8.** Cross-Trait Associations Between Polygenic Scores and Quantitative Symptom Scores Beyond Their Primary Phenotype, Excluding Participants With Any Self-Reported Diagnosis **eTable 9.** Self-Reported Diagnoses Count Among Individuals in the Top 1% of Each Polygenic Score **eTable 10.** Logistic Regression Analyses of Polygenic Score Associations With Risk Group Status Based on Clinical Cutoffs **eTable 11.** Logistic Regression Analyses of Associations Between Polygenic Scores and Top vs Bottom Deciles of Each Symptom Score Distribution

Supplement 3.
Data Sharing Statement

## References

[1] Plomin R. Celebrating a century of research in behavioral genetics. Behav Genet. 2023;53(2):75-84. doi:10.1007/s10519-023-10132-3 36662387 PMC9922236

[2] Abdellaoui A, Yengo L, Verweij KJH, Visscher PM. 15 years of GWAS discovery: realizing the promise. Am J Hum Genet. 2023;110(2):179-194. doi:10.1016/j.ajhg.2022.12.011 36634672 PMC9943775

[3] Wray NR, Lin T, Austin J, . From basic science to clinical application of polygenic risk scores: a primer. JAMA Psychiatry. 2021;78(1):101-109. doi:10.1001/jamapsychiatry.2020.3049 32997097

[4] Ikeda M, Saito T, Kanazawa T, Iwata N. Polygenic risk score as clinical utility in psychiatry: a clinical viewpoint. J Hum Genet. 2021;66(1):53-60. doi:10.1038/s10038-020-0814-y 32770057

[5] Anttila V, Bulik-Sullivan B, Finucane HK, ; Brainstorm Consortium. Analysis of shared heritability in common disorders of the brain. Science. 2018;360(6395):eaap8757. doi:10.1126/science.aap8757 29930110 PMC6097237

[6] Bulik-Sullivan BK, Loh PR, Finucane HK, ; Schizophrenia Working Group of the Psychiatric Genomics Consortium. LD Score regression distinguishes confounding from polygenicity in genome-wide association studies. Nat Genet. 2015;47(3):291-295. doi:10.1038/ng.3211 25642630 PMC4495769

[7] Lee PH, Anttila V, Won H, ; Cross-Disorder Group of the Psychiatric Genomics Consortium. Genomic relationships, novel loci, and pleiotropic mechanisms across eight psychiatric disorders. Cell. 2019;179(7):1469-1482.e11. doi:10.1016/j.cell.2019.11.020 31835028 PMC7077032

[8] Caspi A, Houts RM, Belsky DW, . The p factor: one general psychopathology factor in the structure of psychiatric disorders? Clin Psychol Sci. 2014;2(2):119-137. doi:10.1177/2167702613497473 25360393 PMC4209412

[9] Caspi A, Moffitt TE. All for one and one for all: mental disorders in one dimension. Am J Psychiatry. 2018;175(9):831-844. doi:10.1176/appi.ajp.2018.17121383 29621902 PMC6120790

[10] Lahey BB, Applegate B, Hakes JK, Zald DH, Hariri AR, Rathouz PJ. Is there a general factor of prevalent psychopathology during adulthood? J Abnorm Psychol. 2012;121(4):971-977. doi:10.1037/a0028355 22845652 PMC4134439

[11] Allegrini AG, Cheesman R, Rimfeld K, . The p factor: genetic analyses support a general dimension of psychopathology in childhood and adolescence. J Child Psychol Psychiatry. 2020;61(1):30-39. doi:10.1111/jcpp.13113 31541466 PMC6906245

[12] Grotzinger AD, Mallard TT, Akingbuwa WA, ; iPSYCH; Tourette Syndrome and Obsessive Compulsive Disorder Working Group of the Psychiatric Genetics Consortium; Bipolar Disorder Working Group of the Psychiatric Genetics Consortium; Major Depressive Disorder Working Group of the Psychiatric Genetics Consortium; Schizophrenia Working Group of the Psychiatric Genetics Consortium. Genetic architecture of 11 major psychiatric disorders at biobehavioral, functional genomic and molecular genetic levels of analysis. Nat Genet. 2022;54(5):548-559. doi:10.1038/s41588-022-01057-4 35513722 PMC9117465

[13] Keser E, Liao W, Allegrini AG, . Isolating transdiagnostic effects reveals specific genetic profiles in psychiatric disorders. medRxiv. Preprint posted online April 11, 2024. doi:10.1101/2023.12.20.23300292 PMC13338967 42416636

[14] Selzam S, Coleman JRI, Caspi A, Moffitt TE, Plomin R. A polygenic p factor for major psychiatric disorders. Transl Psychiatry. 2018;8(1):205. doi:10.1038/s41398-018-0217-4 30279410 PMC6168558

[15] Grotzinger AD, Rhemtulla M, de Vlaming R, . Genomic structural equation modelling provides insights into the multivariate genetic architecture of complex traits. Nat Hum Behav. 2019;3(5):513-525. doi:10.1038/s41562-019-0566-x 30962613 PMC6520146

[16] Keser E, Liao W, Allegrini A, . Using polygenic scores corrected for the general psychopathology factor to predict specific psychopathology. Center for Open Science. 2024. Accessed March 25, 2025. http://OSF.IO/29XYT

[17] Lockhart C, Bright J, Ahmadzadeh Y, . Twins Early Development Study (TEDS): a genetically sensitive investigation of mental health outcomes in the mid-twenties. JCPP Adv. 2023;3(2):e12154. doi:10.1002/jcv2.12154 37753150 PMC10519737

[18] Rimfeld K, Malanchini M, Spargo T, . Twins Early Development Study: a genetically sensitive investigation into behavioral and cognitive development from infancy to emerging adulthood. Twin Res Hum Genet. 2019;22(6):508-513. doi:10.1017/thg.2019.56 31544730 PMC7056571

[19] Krapohl E, Patel H, Newhouse S, . Multi-polygenic score approach to trait prediction. Mol Psychiatry. 2018;23(5):1368-1374. doi:10.1038/mp.2017.163 28785111 PMC5681246

[20] Lebeau RT, Glenn DE, Hanover LN, Beesdo-Baum K, Wittchen HU, Craske MG. A dimensional approach to measuring anxiety for DSM-5. Int J Methods Psychiatr Res. 2012;21(4):258-272. doi:10.1002/mpr.1369 23148016 PMC6878356

[21] Conners CK. Conners 3rd edition. MHS Assessments. 2008. Accessed June 3, 2025. https://www.pearsonclinical.co.uk/en-gb/Store/Professional-Assessments/Behaviour/Comprehensive/Conners-3rd-Edition/p/P100009070

[22] Eriksson JM, Andersen LM, Bejerot S. RAADS-14 Screen: validity of a screening tool for autism spectrum disorder in an adult psychiatric population. Mol Autism. 2013;4(1):49. doi:10.1186/2040-2392-4-49 24321513 PMC3907126

[23] Angold A, Costello E, Messer S, Pickles A, Winder F, Silver D. The development of a questionnaire for use in epidemiological studies of depression in children and adolescents. Int J Methods Psychiatr Res. 1995;5:237-249.

[24] Hirschfeld RMA, Williams JBW, Spitzer RL, . Development and validation of a screening instrument for bipolar spectrum disorder: the Mood Disorder Questionnaire. Am J Psychiatry. 2000;157(11):1873-1875. doi:10.1176/appi.ajp.157.11.1873 11058490

[25] Saunders JB, Aasland OG, Babor TF, de la Fuente JR, Grant M. Development of the Alcohol Use Disorders Identification Test (AUDIT): WHO Collaborative Project on Early Detection of Persons with Harmful Alcohol Consumption–II. Addiction. 1993;88(6):791-804. doi:10.1111/j.1360-0443.1993.tb02093.x 8329970

[26] Weathers F, Litz B, Herman D, Huska J, Keane T. The PTSD Checklist (PCL): reliability, validity, and diagnostic utility. In: *Proceedings of the 9th Annual Convention of the International Society for Traumatic Stress Studies*. International Society for Traumatic Stress Studies; 1993.

[27] Ronald A, Sieradzka D, Cardno AG, Haworth CMA, McGuire P, Freeman D. Characterization of psychotic experiences in adolescence using the specific psychotic experiences questionnaire: findings from a study of 5000 16-year-old twins. Schizophr Bull. 2014;40(4):868-877. doi:10.1093/schbul/sbt106 24062593 PMC4059437

[28] Demontis D, Walters GB, Athanasiadis G, ; ADHD Working Group of the Psychiatric Genomics Consortium; iPSYCH-Broad Consortium. Genome-wide analyses of ADHD identify 27 risk loci, refine the genetic architecture and implicate several cognitive domains. Nat Genet. 2023;55(2):198-208. doi:10.1038/s41588-022-01285-8 36702997 PMC10914347

[29] Trubetskoy V, Pardiñas AF, Qi T, ; Indonesia Schizophrenia Consortium; PsychENCODE; Psychosis Endophenotypes International Consortium; SynGO Consortium; Schizophrenia Working Group of the Psychiatric Genomics Consortium. Mapping genomic loci implicates genes and synaptic biology in schizophrenia. Nature. 2022;604(7906):502-508. doi:10.1038/s41586-022-04434-5 35396580 PMC9392466

[30] Purves KL, Coleman JRI, Meier SM, . A major role for common genetic variation in anxiety disorders. Mol Psychiatry. 2020;25(12):3292-3303. doi:10.1038/s41380-019-0559-1 31748690 PMC7237282

[31] Grove J, Ripke S, Als TD, ; Autism Spectrum Disorder Working Group of the Psychiatric Genomics Consortium; BUPGEN; Major Depressive Disorder Working Group of the Psychiatric Genomics Consortium; 23andMe Research Team. Identification of common genetic risk variants for autism spectrum disorder. Nat Genet. 2019;51(3):431-444. doi:10.1038/s41588-019-0344-8 30804558 PMC6454898

[32] Mullins N, Forstner AJ, O’Connell KS, ; HUNT All-In Psychiatry. Genome-wide association study of more than 40,000 bipolar disorder cases provides new insights into the underlying biology. Nat Genet. 2021;53(6):817-829. doi:10.1038/s41588-021-00857-4 34002096 PMC8192451

[33] Howard DM, Adams MJ, Clarke TK, ; 23andMe Research Team; Major Depressive Disorder Working Group of the Psychiatric Genomics Consortium. Genome-wide meta-analysis of depression identifies 102 independent variants and highlights the importance of the prefrontal brain regions. Nat Neurosci. 2019;22(3):343-352. doi:10.1038/s41593-018-0326-7 30718901 PMC6522363

[34] Nievergelt CM, Maihofer AX, Klengel T, . International meta-analysis of PTSD genome-wide association studies identifies sex- and ancestry-specific genetic risk loci. Nat Commun. 2019;10(1):4558. doi:10.1038/s41467-019-12576-w 31594949 PMC6783435

[35] Yu D, Sul JH, Tsetsos F, ; Tourette Association of America International Consortium for Genetics; the Gilles de la Tourette GWAS Replication Initiative; Tourette International Collaborative Genetics Study; Psychiatric Genomics Consortium Tourette Syndrome Working Group. Interrogating the genetic determinants of Tourette’s syndrome and other tic disorders through genome-wide association studies. Am J Psychiatry. 2019;176(3):217-227. doi:10.1176/appi.ajp.2018.18070857 30818990 PMC6677250

[36] Mallard TT, Savage JE, Johnson EC, . Item-level genome-wide association study of the alcohol use disorders identification test in three population-based cohorts. Am J Psychiatry. 2022;179(1):58-70. doi:10.1176/appi.ajp.2020.20091390 33985350 PMC9272895

[37] International Obsessive Compulsive Disorder Foundation Genetics Collaborative (IOCDF-GC) and OCD Collaborative Genetics Association Studies (OCGAS). Revealing the complex genetic architecture of obsessive-compulsive disorder using meta-analysis. Mol Psychiatry. 2018;23(5):1181-1188. doi:10.1038/mp.2017.154 28761083 PMC6660151

[38] Watson HJ, Yilmaz Z, Thornton LM, ; Anorexia Nervosa Genetics Initiative; Eating Disorders Working Group of the Psychiatric Genomics Consortium. Genome-wide association study identifies eight risk loci and implicates metabo-psychiatric origins for anorexia nervosa. Nat Genet. 2019;51(8):1207-1214. doi:10.1038/s41588-019-0439-2 31308545 PMC6779477

[39] Privé F, Arbel J, Vilhjálmsson BJ. LDpred2: better, faster, stronger. Bioinformatics. 2021;36(22-23):5424-5431. doi:10.1093/bioinformatics/btaa1029 33326037 PMC8016455

[40] Privé F, Aschard H, Carmi S, . Portability of 245 polygenic scores when derived from the UK Biobank and applied to 9 ancestry groups from the same cohort. Am J Hum Genet. 2022;109(1):12-23. doi:10.1016/j.ajhg.2021.11.008 34995502 PMC8764121

[41] Carey VJ. gee: Generalized estimation equation solver. The Comprehensive R Archive Network. 2024. Accessed June 28, 2025. https://CRAN.R-project.org/package=gee

[42] Benjamini Y, Hochberg Y. Controlling the false discovery rate: a practical and powerful approach to multiple testing. J R Stat Soc B. 1995;57(1):289-300. doi:10.1111/j.2517-6161.1995.tb02031.x

[43] Waszczuk MA, Jonas KG, Bornovalova M, . Dimensional and transdiagnostic phenotypes in psychiatric genome-wide association studies. Mol Psychiatry. 2023;28(12):4943-4953. doi:10.1038/s41380-023-02142-8 37402851 PMC10764644

[44] Cai N, Revez JA, Adams MJ, ; MDD Working Group of the Psychiatric Genomics Consortium. Minimal phenotyping yields genome-wide association signals of low specificity for major depression. Nat Genet. 2020;52(4):437-447. doi:10.1038/s41588-020-0594-5 32231276 PMC7906795

[45] Stein MB, Levey DF, Cheng Z, ; Department of Veterans Affairs Cooperative Studies Program (no. 575B); VA Million Veteran Program. Genome-wide association analyses of post-traumatic stress disorder and its symptom subdomains in the Million Veteran Program. Nat Genet. 2021;53(2):174-184. doi:10.1038/s41588-020-00767-x 33510476 PMC7972521

[46] Peyrot WJ, Panagiotaropoulou G, Olde Loohuis LM, . Distinguishing different psychiatric disorders using DDx-PRS. medRxiv. Preprint posted online February 4, 2024. doi:10.1101/2024.02.02.24302228 PMC13559851 42625058

[47] Peyrot WJ, Price AL. Identifying loci with different allele frequencies among cases of eight psychiatric disorders using CC-GWAS. Nat Genet. 2021;53(4):445-454. doi:10.1038/s41588-021-00787-1 33686288 PMC8038973

[48] Jones HJ, Heron J, Hammerton G, ; 23 and Me Research Team. Investigating the genetic architecture of general and specific psychopathology in adolescence. Transl Psychiatry. 2018;8(1):145. doi:10.1038/s41398-018-0204-9 30089819 PMC6082910

[49] Neumann A, Jolicoeur-Martineau A, Szekely E, . Combined polygenic risk scores of different psychiatric traits predict general and specific psychopathology in childhood. J Child Psychol Psychiatry. 2022;63(6):636-645. doi:10.1111/jcpp.13501 34389974 PMC9291767

[50] Waszczuk MA, Miao J, Docherty AR, . General *v*. specific vulnerabilities: polygenic risk scores and higher-order psychopathology dimensions in the Adolescent Brain Cognitive Development (ABCD) Study. Psychol Med. 2023;53(5):1937-1946. doi:10.1017/S0033291721003639 37310323 PMC10958676

[51] Cuthbert BN. The RDoC framework: facilitating transition from ICD/DSM to dimensional approaches that integrate neuroscience and psychopathology. World Psychiatry. 2014;13(1):28-35. doi:10.1002/wps.20087 24497240 PMC3918011

[52] Kotov R, Krueger RF, Watson D, . The Hierarchical Taxonomy of Psychopathology (HiTOP): a dimensional alternative to traditional nosologies. J Abnorm Psychol. 2017;126(4):454-477. doi:10.1037/abn0000258 28333488

[53] McGorry PD, Hartmann JA, Spooner R, Nelson B. Beyond the “at risk mental state” concept: transitioning to transdiagnostic psychiatry. World Psychiatry. 2018;17(2):133-142. doi:10.1002/wps.20514 29856558 PMC5980504

[54] Insel T, Cuthbert B, Garvey M, . Research domain criteria (RDoC): toward a new classification framework for research on mental disorders. Am J Psychiatry. 2010;167(7):748-751. doi:10.1176/appi.ajp.2010.09091379 20595427

[55] Woodward DJ, Thorp JG, Middeldorp CM, Akóṣílè W, Derks EM, Gerring ZF. Leveraging pleiotropy for the improved treatment of psychiatric disorders. Mol Psychiatry. 2025;30(2):705-721. doi:10.1038/s41380-024-02771-7 39390223 PMC11746150

[56] Cai N, Verhulst B, Andreassen OA, . Assessment and ascertainment in psychiatric molecular genetics: challenges and opportunities for cross-disorder research. Mol Psychiatry. 2025;30(4):1627-1638. doi:10.1038/s41380-024-02878-x 39730880 PMC11919726

[57] Garcia-Mondragon L, Konac D, Newbury JB, . Role of polygenic and environmental factors in the co-occurrence of depression and psychosis symptoms: a network analysis. Transl Psychiatry. 2022;12(1):259. doi:10.1038/s41398-022-02022-9 35732632 PMC9217963

[58] Piazza GG, Allegrini AG, Eley TC, . Polygenic scores and networks of psychopathology symptoms. JAMA Psychiatry. 2024;81(9):902-910. doi:10.1001/jamapsychiatry.2024.1403 38865107 PMC11170456

[59] van Loo HM, Aggen SH, Kendler KS. The structure of the symptoms of major depression: factor analysis of a lifetime worst episode of depressive symptoms in a large general population sample. J Affect Disord. 2022;307:115-124. doi:10.1016/j.jad.2022.03.064 35367501 PMC10833125

[60] Watts AL, Lane SP, Bonifay W, Steinley D, Meyer FAC. Building theories on top of, and not independent of, statistical models: the case of the *p*-factor. Psychol Inq. 2020;31(4):310-320. doi:10.1080/1047840X.2020.1853476 33510565 PMC7839945

[61] Dahl A, Thompson M, An U, . Phenotype integration improves power and preserves specificity in biobank-based genetic studies of major depressive disorder. Nat Genet. 2023;55(12):2082-2093. doi:10.1038/s41588-023-01559-9 37985818 PMC10703686

[62] Lee PH, Feng YA, Smoller JW. Pleiotropy and cross-disorder genetics among psychiatric disorders. Biol Psychiatry. 2021;89(1):20-31. doi:10.1016/j.biopsych.2020.09.026 33131714 PMC7898275

