# Supplementary material for: Specificity of Polygenic Scores for Psychiatric Disorders Beyond Transdiagnostic Genetic Risk
Source: JAMA Netw Open. 2026 Jan 8;9(1):e2548518. doi: 10.1001/jamanetworkopen.2025.48518 (PMC12784231; doi:10.1001/jamanetworkopen.2025.48518)
Supplement: Supplement 1. — eMethods. eFigure 1. Isolating Transdiagnostic Genetic Effects Across 11 Psychiatric Disorders eResults. eFigure 2. Heat Map of Standardized Symptom Scores in the Top 1% of Each Polygenic Score Distribution eFigure 3. Odds Ratios (ORs) for High-Risk Group Status by Polygenic Scores eFigure 4. Odds Ratios (ORs) for Top vs Bottom Symptom Deciles by Polygenic Score eReferences. [file jamanetwopen-e2548518-s001.pdf]

Supplemental Online Content

Keser E, Liao W, Allegrini AG, et al. Specificity of polygenic scores for psychiatric disorders beyond transdiagnostic genetic risk. *JAMA Netw Open*. 2026;9(1):e2548518.
doi:10.1001/jamanetworkopen.2025.48518

eMethods

eFigure 1. Isolating Transdiagnostic Genetic Effects Across 11 Psychiatric Disorders

eResults

eFigure 2. Heat Map of Standardized Symptom Scores in the Top 1% of Each Polygenic Score Distribution

eFigure 3. Odds Ratios (ORs) for High-Risk Group Status by Polygenic Scores

eFigure 4. Odds Ratios (ORs) for Top vs Bottom Symptom Deciles by Polygenic Score

eReferences

This supplemental material has been provided by the authors to give readers additional information about their work.

© 2026 Keser E et al. *JAMA Network Open*.

eMethods

Psychopathology measures
Questionnaires used to measure symptoms associated with psychiatric disorders. The questionnaires are described in further detail in the TEDS data dictionary (https://www.teds.ac.uk/datadictionary/home.htm).

Anxiety symptoms:
Anxiety symptoms were assessed using the Generalised Anxiety Disorder – Dimensional (GAD-D) scale [1]. This questionnaire comprises of 10 items that measure thoughts, feelings, and behaviours commonly associated with worry about everyday concerns such as family, health, finances, school, and work. Each item is rated on a 5-point scale: "Never" (0), "Occasionally" (1), "Half of the time" (2), "Most of the time" (3), "All of the time" (4). Total scores range from 0 to 40, with higher scores indicating greater levels of anxiety.

Attention Deficit Hyperactivity Disorder (ADHD) symptoms:
ADHD symptoms were measured using the Conners-3-Self-Report,[2] which assesses ADHD symptoms in adults. In TEDS, only the 11 items from the inattention subscale were administered. These items measure difficulties such as poor concentration, distractibility, and challenges with initiating or completing tasks. Each item is rated on a 4-point scale: "Not true at all" (0), "Somewhat true" (1), "Mainly true" (2), and "Definitely true" (3). Total scores range from 0 to 33, with higher scores indicating greater levels of attention difficulties.

Alcohol use:
Alcohol use was assessed using the adapted version of the Alcohol Use Disorders Identification Test (AUDIT),[3] a 10-item screening tool used to measure alcohol consumption patterns, dependence symptoms, and harmful consequences of alcohol use. The first three items assess general consumption (i.e., quantity and frequency of alcohol use). The items 4-10 assess problematic drinking behaviour and adverse outcomes. Each item is rated on a 5-point scale (0-4) indicating the frequency or severity of the behaviour. Total scores range from 0 to 40, with higher scores indicating more problematic or risky alcohol use.

Autism Spectrum Disorder (ASD) traits:
Autistic traits were assessed using a shortened version of the Ritvo Autism and Asperger Diagnostic Scale (RAADS-14),[4] a screening tool for ASD in adults. In TEDS, a 6-item version of the questionnaire was administered, comprising three socio-communicative and three non-social items assessing behaviours related to social interaction, communication, and sensory sensitivity characteristic of ASD. Each item is rated on a 4-point scale: "True now and when I was young" (0), "True only now" (1), "True only when I was younger than 17" (2), and "Never true" (3). After reverse-coding, total scores range from 0 to 18, with higher scores indicating more pronounced autistic traits.

Depression symptoms:
Depression symptoms were assessed using the Short Mood and Feelings Questionnaire (MFQ-13),[5] a 13-item measure designed to capture affective, cognitive, and behavioural symptoms of depression experienced over the preceding two weeks. Each item is rated on a 3-point scale: "Not true" (0), "Sometimes true" (1), or "True" (2). Total scores range from 0 to 26, with higher scores suggesting more severe depressive symptoms.

Mania/Hypomania symptoms:
Symptoms of mania and hypomania were assessed using the Mood Disorder Questionnaire (MDQ),[6] a screening measure for bipolar disorder. The MDQ includes 13 yes/no items assessing experiences consistent with DSM-IV [7] criteria for manic or hypomanic episodes (e.g., elevated mood, increased activity, decreased need for sleep). Additional items assess whether symptoms occurred during the same time period and whether they caused functional impairment. The total symptom score is calculated as the sum of endorsed items, ranging from 0 to 13, with higher scores indicating greater manic or hypomanic symptomatology.

Post-Traumatic Stress Disorder (PTSD) symptoms:
PTSD symptoms were assessed using the Post-Traumatic Stress Disorder Checklist (PCL-6),[8] a validated 6-item short form of the original 17-item PCL.[9] The PCL-6 captures the main DSM-V[10] symptom clusters: re-experiencing, avoidance, negative alterations in cognition and mood, and hyperarousal. In TEDS, participants rated how much each symptom had bothered them in relation to *any* stressful experience. Items are rated on a 5-point scale: "Not at all" (0), "A little bit" (1), "Moderately" (2), "Quite a bit" (3), "Extremely" (4). Total scores range from 0 to 24, with higher scores indicating greater PTSD symptom severity.

© 2026 Keser E et al. *JAMA Network Open*.

Psychotic symptoms:
Psychotic-like experiences were assessed using the Specific Psychotic Experiences Questionnaire (SPEQ),[11] a measure assessing symptoms of hallucination, paranoia, cognitive disorganization, grandiosity, anhedonia, and negative symptoms. In TEDS, data were collected for the paranoia (15 items) and hallucinations (9 items) subscales. Items are rated on a 7-point scale: "Not at all" (0), "Rarely" (1), "Once a month" (2), "Once a week" (3), "Several times a week" (4), and "Daily" (5). Total scores range from 0 to 75 for the paranoia subscale and from 0 to 54 for the hallucinations subscale, with higher scores suggesting more frequent or severe psychotic-like experiences.

Construction of the genomic p-factor
Genomic structural equation modelling (SEM) is a framework that applies structural equation modelling to genome-wide association study (GWAS) summary statistics to model patterns of genetic correlations between complex traits. We used genomic SEM to perform multivariate genome-wide association analysis of 11 major psychiatric disorders in order to capture genetic effects shared across disorders (p) and residual-disorder specific effects (non-p).

GWAS summary statistics for the eleven disorders were formatted using the *munge* function in genomic SEM R package v.0.0.5 (using default parameters). The *munge* function converts the summary statistics to the format expected by linkage disequilibrium (LD) score regression, restricting SNPs to those present in HapMap3 with a minor allele frequency > 1% and information score > 0.9. The LD weights used for LD score regression were calculated using the European subsample of the 1000 Genomes phase 3 project; excluding the major histocompatibility complex (MHC) due to complex LD structures in this region that can bias estimates. After the quality control steps, 3,746,806 SNPs were present across all 11 disorders.

The processed summary statistics were then used in a multivariable LD score regression in genomic SEM to estimate the genetic covariance and sampling covariance matrices. These were transformed into genetic correlation and sampling correlation matrices for model fitting.

A common factor was fitted, with all disorders loading on one latent dimension (i.e., the p factor; eFigure 1). Residual variance for each disorder was simultaneously estimated to capture disorder-specific signal (non-p). We ran a GWAS on p and residual variance in each psychiatric disorder. We repeated this procedure 11 times to isolate transdiagnostic genetic effects from each of the 11 major psychiatric disorders.

In deriving the p factor, we evaluated the genetic correlation matrix for near-singularity and confirmed that estimation proceeded without convergence warnings. All factor loadings were interpretable and consistent with prior research on transdiagnostic liability. To estimate the p factor, we compared two estimators: diagonally weighted least squares (DWLS) which allowed trait loadings to vary freely, and maximum likelihood (ML) which accounted for differences in GWAS sample sizes. Although both models produced similar loadings, ML yielded poorer model fit, supporting our use of DWLS. The model provided adequate fit (χ^2 (44) = 950.4836, AIC = 994.48, CFI = .82, SRMR = .12).

© 2026 Keser E et al. *JAMA Network Open*.

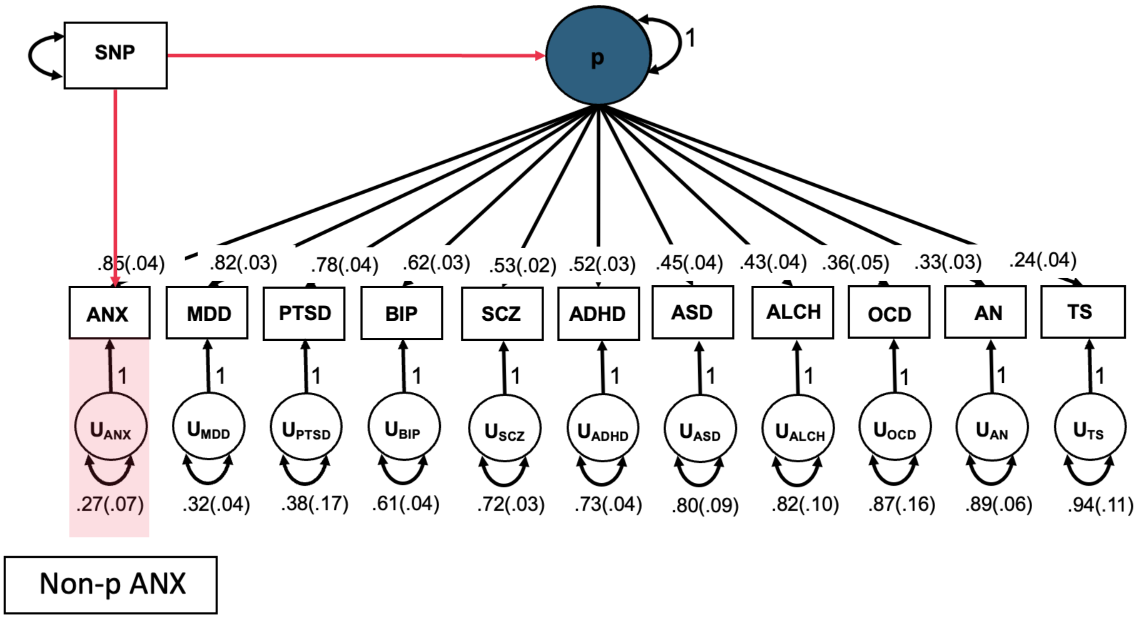

eFigure 1. Isolating Transdiagnostic Genetic Effects Across 11 Psychiatric Disorders. Standardized results from a common factor model of genomic p. Squares represent observed variables (GWAS summary statistics) and circles represent latent variables that are statistically inferred from the data (for ex., genomic p-factor). One-headed arrows show standardized factor loadings, representing regression relations with the arrow pointing from the predictor variable to the outcome variable. Covariance relationships between variables are represented as two-headed arrows linking the variables. Residual variances of a variable are represented as a two-headed arrow connecting the variable to itself. Standard errors are in parentheses. The red arrows linking the SNP to both the p-factor and ANX provide an example of the model used to partition genetic variance associated with transdiagnostic effects from the genetic variance specific to each disorder. This model was repeated for each disorder. ANX = anxiety disorder; MDD = major depressive disorder; PTSD = post-traumatic stress disorder; BIP = bipolar disorder; SCZ = schizophrenia; ADHD = attention-deficit hyperactivity disorder; ASD = autism spectrum disorder; ALCH = problematic alcohol use; OCD = obsessive-compulsive disorder; AN = anorexia nervosa; TS = Tourette syndrome; p = general psychopathology factor.

Sensitivity analyses

We conducted a series of sensitivity analyses to ensure robustness of the primary findings. First, to assess what the p PGS capture phenotypically in the current sample, we examined whether individuals in the top 1% of the p PGS distribution showed distinct patterns of symptom severity and diagnostic burden compared with those in the top 1% of each uncorrected and non-p PGS. We also examined self-reported diagnoses and comorbidity patterns.

Second, we assessed the clinical relevance of PGSs, we compared associations across the p PGS, uncorrected PGSs, and non-p PGSs using two complementary approaches. First, we defined binary high-risk groups for each of the symptom score distribution using either established clinical cutoffs (for MDD,[12,13] ANX,[14] PTSD,[15] ALCH,[3] and hypomania) or the top 5% of the distribution (for ADHD, ASD) when clinical cutoffs were not available. The remaining sample served as the control group. Next, to evaluate performance across the full spectrum of severity, we also compared individuals in the top versus bottom 10% of each quantitative symptom score distribution.

For both analyses, logistic regressions were conducted using a generalized estimating equations (GEE) framework. All continuous variables were residualized for age and sex, and the residuals were standardized prior to analyses. All models included the same covariates as in the primary analyses (i.e., top 10 ancestry PCs, genotyping batch, and chip). Effect sizes are presented as adjusted odds ratios (ORs) per standard deviation (SD), with 95% confidence intervals.

© 2026 Keser E et al. *JAMA Network Open*.

eResults

Sensitivity analyses

Phenotypic profile of individuals in the top 1% of the p PGS

Individuals in the top 1% of the p PGS exhibited elevated symptom levels across nearly all psychiatric domains, consistent with the interpretation of the p as an index of transdiagnostic liability (eFigure 2). In contrast, those in the top 1% of uncorrected PGSs showed more heterogeneous elevations across symptom profiles, with generally lower symptom elevations. Notable exceptions included the PGSs for ADHD and ALCH PGS, which showed broader symptom elevations relative to other uncorrected scores. Most non-p PGSs yielded average symptom levels close to the sample mean, indicating more limited phenotypic impact.

Rates of self-reported diagnoses were similar across the top 1% of all PGSs. For example, in the top 1% of the p PGS distribution 47 of 51 individuals (92.2%) reported at least one psychiatric diagnosis. The most frequently endorsed conditions were MDD (23 cases; 47.1%), followed by OCD (7 cases; 13.7%), ANX (6 cases; 11.8%), and PTSD (4 cases;7.8%). Results were similar for uncorrected PGSs, including ADHD (49 cases), MDD (40 cases), and ANX (36 cases), and for non-p PGSs, such as ADHD non-p (35 cases). Results are provided in eTable 9 in Supplement 2. A slightly different pattern emerged when examining individuals reported two or more diagnoses: the p (11 individuals), ADHD (13 individuals), and ANX (10 individuals).

It is important to note that the top 1% PGS groups are not independent. For example, an individual with high p PGS and 2+ diagnoses likely also fall into the top 1% for ADHD or ANX. Therefore, these results illustrates the general patterns of psychiatric burden among individuals with high PGSs.

PGS associations with high-risk vs controls

The p PGS was significantly associated with high-risk group across nearly all domains tested (ORs ranging from 1.26 to 1.45; all $p < .001$), with the exception of alcohol use symptoms (OR = 1.11, 95% CI [0.88–1.41]). Uncorrected PGSs were also significantly associated with high-risk status in most domains, with ORs very similar to p PGSs. In most domains, non-p PGSs were not associated with high-risk status. Consistent with primary findings, exceptions included the PTSD non-p PGS (OR = 1.18, 95% CI [1.07–1.30]) and ALCH non-p PGS (OR = 1.44, 95% CI [1.16–1.79]), suggesting retained disorder-specificity. Results are provided in eTable 10 in Supplement 2.

PGS associations with top 10% vs bottom 10% of symptom score distributions

Consistent patterns of associations were observed when comparing individuals in the top versus bottom deciles of symptom severity distributions. The p PGS and uncorrected PGSs showed significant associations across most domains. In addition to non-p PGS for PTSD and ALCH, non-p ADHD also showed a modest but significant association with extremes (OR = 1.18, 95% CI, 1.03–1.34) were significantly associated with symptom extremes. Results are provided in eTable 11 in Supplement 2.

© 2026 Keser E et al. *JAMA Network Open*.

eFigures

eFigure 2. Heat Map of Standardized Symptom Scores in the Top 1% of Each Polygenic Score Distribution. Rows represent types of PGS, columns represent symptom domains. Colour intensity reflects the mean standardized symptom score within each top 1% polygenic score group, with red indicating above-average symptom levels and blue indicating below-average levels. BIP= Bipolar disorder; MDD= Major depressive disorder; SCZ= Schizophrenia; ASD= Autism-spectrum disorder; ANX= Anxiety disorder; PTSD= Post-traumatic stress disorder; ALCH= Problematic alcohol use.

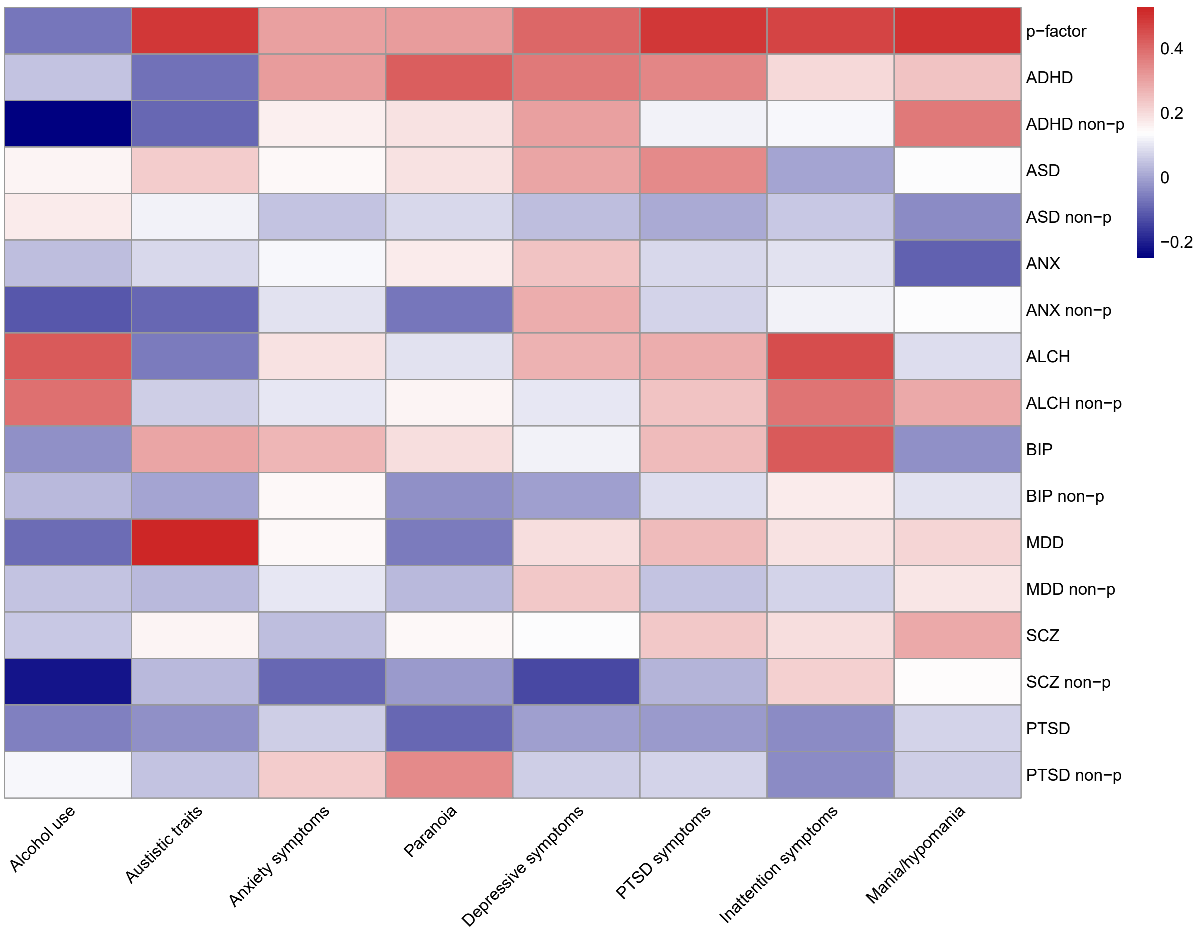

© 2026 Keser E et al. *JAMA Network Open*.

eFigure 3. Odds Ratios (ORs) for High-Risk Group Status by Polygenic Scores. Each point represents the odds ratio (OR) for being in the high-risk group, as estimated from logistic regression models within generalized estimating equations. Models were run separately for each of three polygenic scores: transdiagnostic (p), uncorrected, and p-corrected (non-p). All models included the first 10 principal components, genotyping batch, and chip as covariates. Error bars indicate 95% confidence intervals. The dashed line indicates the null value (OR = 1). The x-axis is plotted on a log scale.

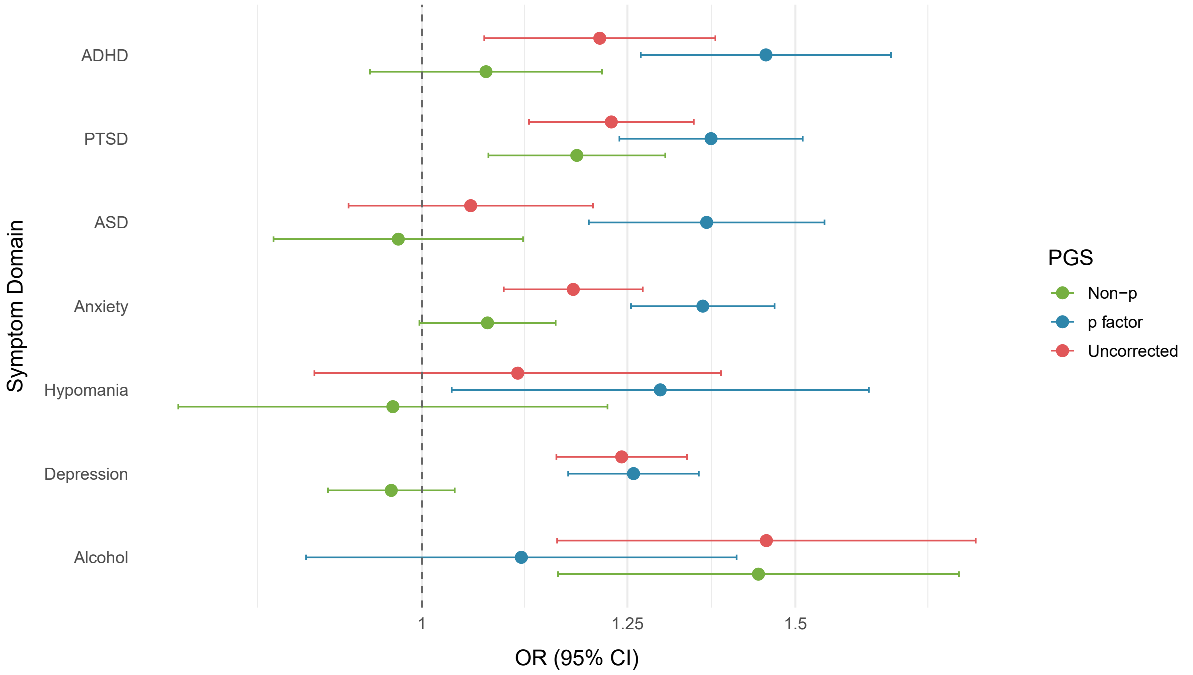

© 2026 Keser E et al. *JAMA Network Open*.

eFigure 4. Odds Ratios (ORs) for Top vs Bottom Symptom Deciles by Polygenic Score. Each point represents the odds ratio (OR) for being in the top 10% versus bottom 10% of symptom severity, as estimated from logistic regression models within generalized estimating equations. Models were run separately for each of three polygenic scores: transdiagnostic (p), uncorrected, and p-corrected (non-p). All models included the first 10 principal components, genotyping batch, and chip as covariates. Error bars indicate 95% confidence intervals. The dashed line indicates the null value (OR = 1). The x-axis is plotted on a log scale.

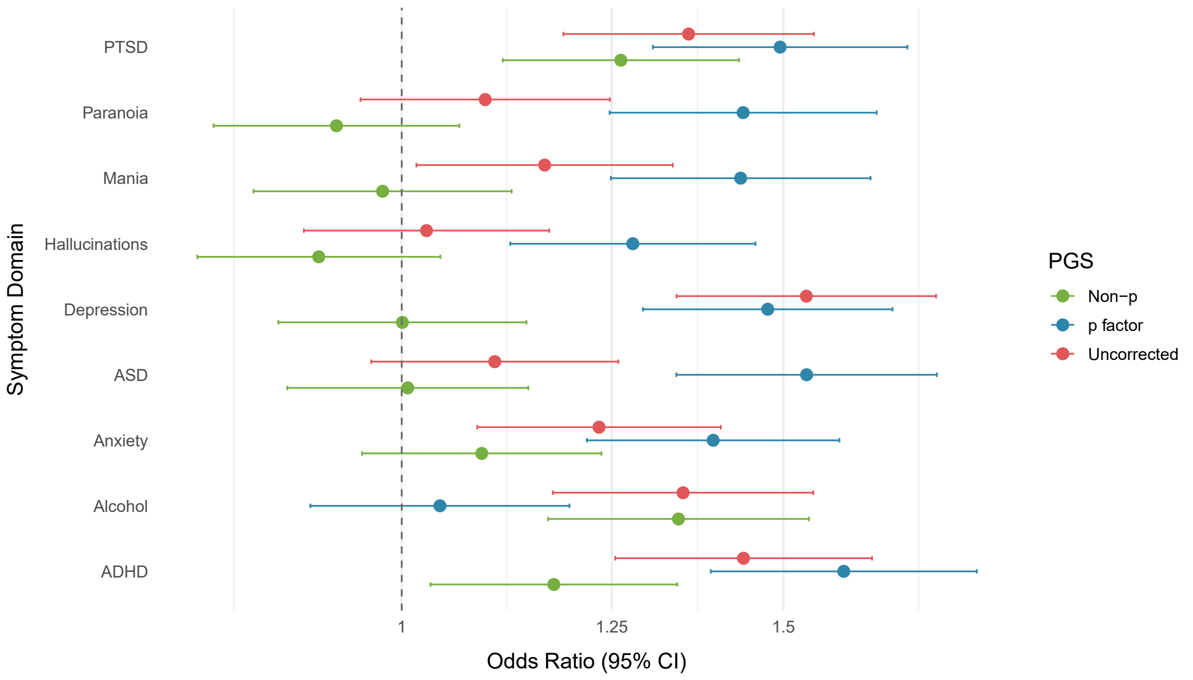

© 2026 Keser E et al. *JAMA Network Open*.

eReferences

1. Lebeau RT, Glenn DE, Hanover LN, Beesdo-Baum K, Wittchen H, Craske MG. A dimensional approach to measuring anxiety for DSM-5. *Int J Methods Psychiatr Res*. 2012;21(4):258-272. doi:10.1002/mpr.1369

2. Conners CK. Conners 3rd Edition (Conners 3) Manual. New York: MHS Assessments. Published online 2008.

3. Saunders JB, Aasland OG, Babor TF, De La Fuente JR, Grant M. Development of the Alcohol Use Disorders Identification Test (AUDIT): WHO Collaborative Project on Early Detection of Persons with Harmful Alcohol Consumption-II. *Addiction*. 1993;88(6):791-804. doi:10.1111/j.1360-0443.1993.tb02093.x

4. Eriksson JM, Andersen LM, Bejerot S. RAADS-14 Screen: validity of a screening tool for autism spectrum disorder in an adult psychiatric population. *Mol Autism*. 2013;4(1):49. doi:10.1186/2040-2392-4-49

5. Angold A, Costello E, Messer S, Pickles A, Winder F, Silver D. The Development of a Questionnaire for Use in Epidemiological Studies of Depression in Children and Adolescents. *Int J Methods Psychiatr Res*. 1995;5:237-249.

6. Hirschfeld RMA, Williams JBW, Spitzer RL, et al. Development and Validation of a Screening Instrument for Bipolar Spectrum Disorder: The Mood Disorder Questionnaire. *Am J Psychiatry*. 2000;157(11):1873-1875. doi:10.1176/appi.ajp.157.11.1873

7. *Diagnostic and Statistical Manual of Mental Disorders, 4th Ed.* American Psychiatric Publishing, Inc.; 1994:xxvii, 886.

8. Lang AJ, Stein MB. An abbreviated PTSD checklist for use as a screening instrument in primary care. *Behav Res Ther*. 2005;43(5):585-594. doi:10.1016/j.brat.2004.04.005

9. Weathers F, Litz B, Herman D, Huska J, Keane T. The PTSD Checklist (PCL): Reliability, Validity, and Diagnostic Utility. Paper presented at the Annual Convention of the International Society for Traumatic Stress Studies, San Antonio, TX. In: ; 1993.

10. *Diagnostic and Statistical Manual of Mental Disorders: DSM-5™, 5th Ed.* American Psychiatric Publishing, Inc.; 2013:xliv, 947. doi:10.1176/appi.books.9780890425596

11. Ronald A, Sieradzka D, Cardno AG, Haworth CMA, McGuire P, Freeman D. Characterization of Psychotic Experiences in Adolescence Using the Specific Psychotic Experiences Questionnaire: Findings From a Study of 5000 16-Year-Old Twins. *Schizophr Bull*. 2014;40(4):868-877. doi:10.1093/schbul/sbt106

12. Eyre O, Bevan Jones R, Agha SS, et al. Validation of the short Mood and Feelings Questionnaire in young adulthood. *J Affect Disord*. 2021;294:883-888. doi:10.1016/j.jad.2021.07.090

13. Thabrew H, Stasiak K, Bavin L, Frampton C, Merry S. Validation of the Mood and Feelings Questionnaire (MFQ) and Short Mood and Feelings Questionnaire (SMFQ) in New Zealand help-seeking adolescents. *Int J Methods Psychiatr Res*. 2018;27(3):e1610. doi:10.1002/mpr.1610

14. Beesdo-Baum K, Klotsche J, Knappe S, et al. PSYCHOMETRIC PROPERTIES OF THE DIMENSIONAL ANXIETY SCALES FOR DSM-V IN AN UNSELECTED SAMPLE OF GERMAN TREATMENT SEEKING PATIENTS: Research Article: Dimensional Anxiety Scales for DSM-V. *Depress Anxiety*. 2012;29(12):1014-1024. doi:10.1002/da.21994

15. Lang AJ, Wilkins K, Roy-Byrne PP, et al. Abbreviated PTSD Checklist (PCL) as a guide to clinical response. *Gen Hosp Psychiatry*. 2012;34(4):332-338. doi:10.1016/j.genhosppsych.2012.02.003

© 2026 Keser E et al. *JAMA Network Open*.
