# Supplementary material for: Specificity of Polygenic Scores for Psychiatric Disorders Beyond Transdiagnostic Genetic Risk
Source: JAMA Netw Open. 2026 Jan 8;9(1):e2548518. doi: 10.1001/jamanetworkopen.2025.48518 (PMC12784231; doi:10.1001/jamanetworkopen.2025.48518)
Supplement: Supplement 3. — Data Sharing Statement [file jamanetwopen-e2548518-s003.pdf]

Data Sharing Statement

Keser. Specificity of Polygenic Scores for Psychiatric Disorders Beyond Transdiagnostic Genetic Risk. *JAMA Netw Open*. Published December 30, 2025.
doi:10.1001/jamanetworkopen.2025.48518

Data
Data available: No

Additional Information
Explanation for why data not available: The GWAS data used in the study are publicly available. The Twins Early Development Study resource is held by King's College London. Data can be made available, subject to a data sharing agreement, as detailed at https://www.teds.ac.uk/researchers/teds-data-access-policy.
